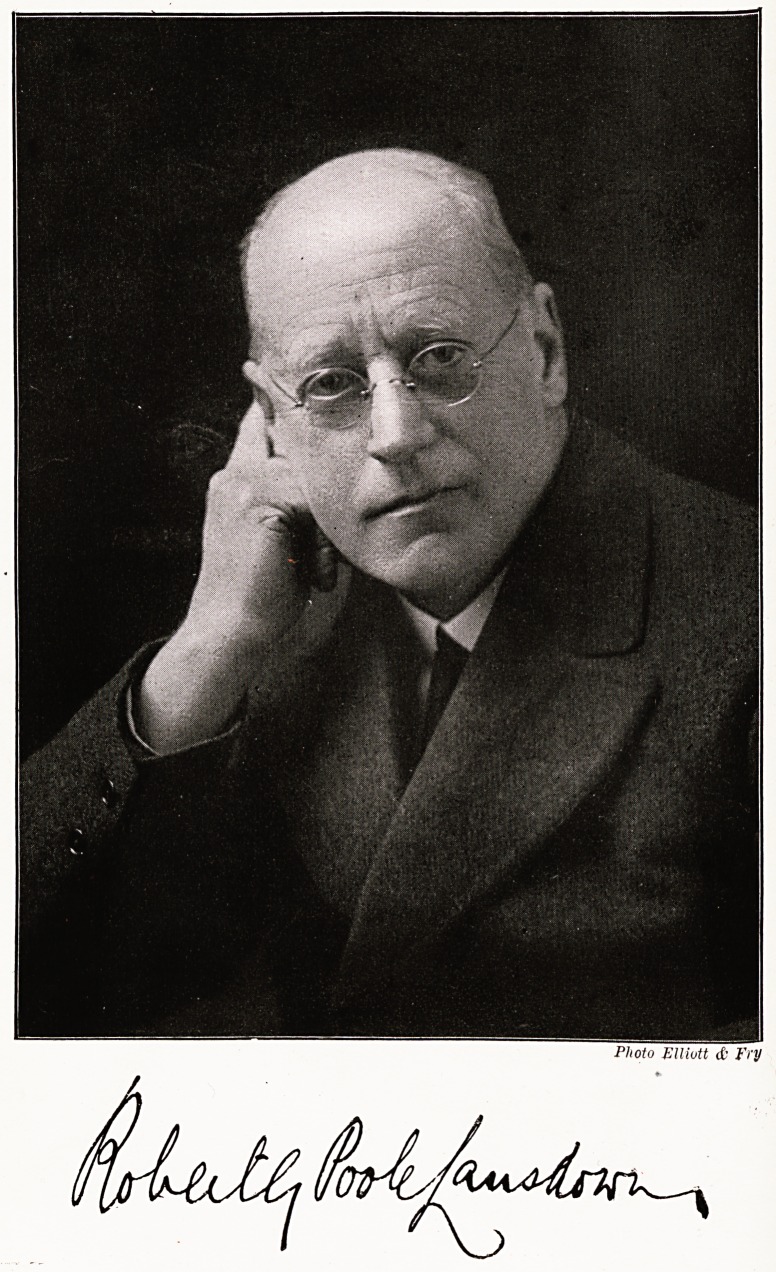# R. G. Poole Lansdown

**Published:** 1924-04

**Authors:** 


Robert G Poole Lansdown

### Obituary.

---

### R. G. POOLE LANSDOWN, M.D., B.S.

It is with deep regret that we have to record the death, on Feb. 17th, at the age of 60, of Mr. Robert Guthrie Poole Lansdown, Consulting Surgeon to the Bristol General Hospital.

He was for a very short time at Epsom College, whence he came to Clifton College in the days of Percival. For a few terms he studied at University College, Bristol, but he was essentially a Guy's Hospital man, and for his Hospital he always retained a loyal affection, and as far as a provincial surgeon can he kept in touch with the staff. His father before him and his own son were also Guy's men, and his son, Dr. R. B. P. Lansdown, has held resident appointments there.

As a student Lansdown had the advantage of boarding with Mr. Jacobson, then an Assistant Surgeon, who coached him for his examinations. Jacobson inspired his pupil with his own enthusiasms, and many were the stories which Lansdown had to tell of this great teacher's acumen and quaint humour.

Graduating in the Durham University M.B. and B.S. in 1889, he also took the Conjoined Diplomas, and in 1891 the Durham M.D.

After holding the appointment of House Surgeon at Guy's he had to return to Bristol to assist in his father's practice. At first he was appointed Anæsthetist to the Bristol General Hospital, where his father and grandfather had been members of the honorary staff.

Chloroform in his hands seemed a safe anæsthetic, and he preferred it for abdominal operations, although he always left the choice of anæsthetic to the administrator. Until quite recent years it was not uncommon to see Lansdown giving the anæsthetic whilst he supervised an operation performed by his House Surgeon. For some years after his appointment as

Assistant Surgeon he continued to assist his father in general practice. This led to his appointment as Medical Officer to the Post Office Staff, and in such high esteem and affection was he held that the older postmen sent a wreath for his funeral, although it must have been nearly twenty years since he resigned.

He was almost the last to combine general practice with the appointment of Hon. Surgeon to the General Hospital, but after a while confined himself to pure consulting surgery, a course that was almost immediately justified. He was quite aware of the value these years of general practice had been to him in his later work, although he often regretted that he had been unable to specialise before.

Lansdown was Lecturer in Practical Surgery in the Bristol University, and was one of the most useful members of the Medical Board, of which for some time he was Chairman. He was also Consulting Surgeon, Clifton Dispensary, and to the Homœopathic Hospital.

During the war he was a Major in the R.A.M.C. (T.), and attached to the Second Southern General Hospital, Bristol, and did much to assist the good-fellowship between the staffs of the Royal Infirmary and the General Hospital. He was given the rank of Lieut.-Colonel just before the end of the war.

As a Surgeon he combined a rare and ripe judgment with a fine technique and skill in operating. To the last he never allowed himself to get into a groove, but was quick to adopt the real advances in his subject. He was among the first to adopt in its entirety the modern methods of aseptic surgery, installing a steam-sterilizer in his own house.

Perhaps the most important of his contributions to the journals on surgery was one which he wrote in conjunction with Dr. G. Scott Williamson on the "Etiology of Appendicitis, Gastric Ulcer and Allied Conditions," in which the primary lesions in these cases were described as being in the lymphoid tissue in the wall of the alimentary canal.

In his work Lansdown had two outstanding characteristics, his warm and understanding humanity towards his patients and his strict and rigid attention to the smallest details.

Brought up in a very strict school of surgery, he held on to the best in the old technique, even to the orthodox method of holding the scalpel and the correctness and beauty of his bandaging. In the councils of his colleagues he held the highest place, and when there was any doubt as to the right thing to do in any difficulty Lansdown was the first to be appealed to.

He was wholely unselfish, warm-hearted, shrewd and absolutely straightforward, and the knowledge that Lansdown never descended to devious diplomacy contributed much to the weight that his opinion carried.

He had been President of the Bristol Medico-Chirurgical Society and President of the Bath and Bristol Branch of the British Medical Association.

For a particularly plucky rescue of a lady from drowning he was awarded the Bronze Medal of the Royal Humane Society.

He had numerous interests outside his profession. He was a very keen motorist, and was looking forward at the time of his death to the delivery of a Lanchester car for which he had been waiting for some time. When it was possible he went abroad for his holidays since his first visit to the Black Forest in 1906. Switzerland was his favourite holiday resort, where he did a good deal of climbing, but he had also spent some time in Italy and the south of France at various times. An excellent photographer with a perfect technique, he had been President of the Bristol and West of England Photographic Society.

There are many who will remember his art as an amateur actor when he took part in the "Medical Dramatics." Amongst his conspicuous successes were the rôles of the title-part in *The Magistrate*, "Pete" in *The Professor's Love Story*, and, where perhaps he was seen at his best, as the "Dean" in *Dandy Dick*.

His dramatic instinct showed itself even when he was repeating an ordinary conversation, for he caught with amazing skill the tone of voice, the peculiarities of speech, and the mannerisms of the person whom he was quoting. It is not so generally known that, although he was not a good public speaker, he was an exceptionally fine reader. He once coached two boys for the Reading Prize at Clifton College. One secured the prize, and in another year the other came second. He was once handed a passage to read from the *Ring and the Book.* Lansdown read it aloud, and continued page after page until he came to the end of the section. It was by no means an easy sample, and he had never seen it before, but not once did he stumble, or so much as trip in bringing out the sense so as to necessitate the re-reading of a single line. It was no mean feat to accomplish.

When he reached the age of sixty he resigned his appointments at the University and from the Bristol General Hospital which he loved so well and so loyally and devotedly had served. He decided to give up his practice in March this year and was taking up new interests to occupy his unaccustomed leisure, when the end came with appalling suddenness from syncope. Yet it was, for himself, the end he desired.

### BIBLIOGRAPHY.

(With J. M. Clarke) "A Case of Sarcoma of the Brain removed by Operation; subsequent Operations for removal of a Second Tumour; Recovery," *Brit. M. J.*, 1901, i. 879–881.

"Pneumococcal Arthritis," *Bris. Med.-Chir. J.*, 1911, xxix.

(With J. M. Clarke) "Intra-Medullary Tumour of the Spinal Cord: Treatment by Laminectomy and Application of Radium," *Brit. M. J.*, 1914, i. 1009.

(With G. Scott Williamson) "The Etiology of Appendicitis, Gastric Ulcer, and Allied Conditions," *Brit. J. Surg.*, 1914–15, ii. 306.

"Removal of Bullets and other Metallic Foreign Bodies," *Bris. Med.-Chir. J.*, 1915, xxxiii. 157–162.

"The Systematic Examination of the Abdomen," *Bris. Med.-Chir. J.*, 1918–19, xxxvi. 33–47: also *Antiseptic*, 1919, xvi. 367–377.

"Report on Surgery," *Bris. Med.-Chir. J.*, 1911, xxix. 73.

## Figures and Tables

**Figure f1:**